# Expression and biological significance of c-FLIP in human hepatocellular carcinomas

**DOI:** 10.1186/1756-9966-28-24

**Published:** 2009-02-20

**Authors:** Xilin Du, Guoqiang Bao, Xianli He, Huadong Zhao, Fang Yu, Qing Qiao, Jianguo Lu, Qingjiu Ma

**Affiliations:** 1 Department of general surgery, Tangdu Hospital, Fourth Military Medical University, Xi'an 710038, PR China; 2 Department of biochemistry, Fourth Military Medical University, Xi'an 710032, PR China

## Abstract

**Background:**

c-FLIP can be considered as a tumor-progression factor in regard to its anti-apoptotic functions. In the present study, we intended to investigate the expression of c-FLIP in human HCC tissues, and its relation with drug-induced cell apoptosis through the specific inhibition of c-FLIP expression by siRNA in 7721 cells.

**Methods:**

c-FLIP expression was quantified immunohistochemically in HCC tissues(eighty-six cases), and corresponding noncancerous tissues (fifty-seven cases). Patients with HCC were followed up for cancer recurrence. Then, the c-FLIP gene was silenced with specific siRNA in 7721 HCC cells. c-FLIP expression was detected by RT-PCR, Western Blot and immunocytochemical staining. The cellular viability and cell apoptosis were assayed *in vitro *with cells treated with doxorubicin.

**Results:**

Positive immunostaining was detected for c-FLIP in 83.72% (72/86) human HCC tissues, 14.81% (4/27) hepatic cirrhosis, 11.11% (2/18) hepatic hemangioma tissues, and absent in normal hepatic tissues. The overexpression(more than 50%) of c-FLIP in HCC adversely affected the recurrence-free survival. Through c-FLIP gene silencing with siRNA, the expressions of c-FLIP mRNA and protein were remarkably down-regulated in 7721 HCC cells. And doxorubicin showed apparent inhibition on cell proliferations, and induced more apoptosis.

**Conclusion:**

These results indicate that c-FLIP is frequently expressed in human HCCs, and its overexpression implied a lesser probability of recurrence-free survival. The specific silencing of c-FLIP gene can apparently up-regulate drug-induced HCC cell apoptosis, and may have therapeutic potential for the treatment of human HCC.

## Background

Human HCC (hepatocellular carcinomas) is the common hepatic highly malignant tumor. Most patients, especially in China, present at diagnosis with a high stage. The etiopathogenisis and developments of HCC are not well known. Deregulation of cell proliferation and cell apoptosis underlies neoplastic initiation and development, which involves multiple gene alterations, and is regulated by complicated signal transduction pathways. It has become clear that deregulated apoptosis plays a pivotal role in tumorigenesis, malignancy and metastatic potential [1].

Accumulating evidence suggests that multiple intrinsic and extrinsic signaling molecules contribute to the resistance to death ligands- and chemotherapeutics-induced apoptosis in cancer cells. c-FLIP(cellular FLICE-inhibitory protein) is a novel member of IAP(inhibitor of apoptosis protein) family, which inhibits the apoptosis signaling mediated by the death receptors Fas, DR4, and DR5[2,3]. c-FLIP plays a pivotal role in modulating the induction of apoptosis in variant cancer cells [4-6]. Down-regulating c-FLIP expression confers sensitivity to TRAIL- and Fas-induced apoptosis.

c-FLIP has homology to caspase-8 and caspase-10, but lacks their protease activity due to the absence of key NH_2 _acid residues at the active site[7]. c-FLIP belongs to the potential negative regulators of the DR(death receptor) pathway by interfering with caspase-8 activation. Two splicing variants of c-FLIP, 55 kDa c-FLIP_L_(long form) and 25 kDa c-FLIP_S_(short form), have the capacity to block DR-mediated apoptosis. c-FLIP_L _and c-FLIP_S _have been described to be recruited to the DISC(death-inducing signaling complex) and prevent caspase-8 activation in slightly different ways. Although the precise physiological role of c-FLIP is still debated, it is generally accepted that c-FLIP_S _interferes with the initial cleavage between the p20 and the p10 subunits of caspase-8, while c-FLIP_L _blocks the final cleavage step between the prodomain and the p20 subunit of the p43/41 intermediate unit. In contrast to c-FLIP_S_, c-FLIP_L _can interact with both FADD and caspase-8, and it has the more potent inhibitory activity and prevents caspase-8 activation by acting as a substrate trap [8-10].

In addition, c-FLIP is a target for the major survival pathways involved in carcinogenesis, namely the NF-κB, Akt/PKB and MAPK pathways [11]. Moreover, c-FLIP conveys independent prognostic information in the presence of classical prognosticators [12].

RNA interference (RNAi) represents a phenomenon of double-stranded RNA (dsRNA)-mediated post-transcriptional gene silencing (PTGS). RNAi can highly induce specific target gene silencing in mammalian cells using small interfering RNA (siRNA) [13]. It has been shown that down-regulation of c-FLIP_L _in many cells by siRNA sensitizes the cells to ligands- and chemotherapeutics-induced apoptosis [14].

In this study, the expression of c-FLIP in human HCC tissues and corresponding noncancerous tissues was analyzed by immunohistochemical staining. And then, the plasmids, which could encode siRNA against c-FLIP, were constructed and transfected into 7721 cells, a typical human HCC cell line, to inhibit the c-FLIP expression for the further study on its biological activity.

## Methods

### Patients and samples

Eighty-six patients with HCC presenting at Tangdu and Xijing Hospital of FMMU between 1999 and 2006, for whom sufficient paraffin embedded tissue was available, were enrolled in the present investigation. All the patients were not given the adjuvant radio- and/or chemo-therapy before the resection. Of the patients, seventy were male and sixteen were female with median age 65 years (range 31 to 76). The mean size of tumor was 5.5 ± 2.1 cm (mean ± SD) in diameter with a range from 2.5 to 11.0 cm(For the patient with multiple focus, the dimension of the largest tumor was recorded). Tumor staging was in accordance to the AJCC staging system. 27 cases of hepatic cirrhosis, eighteen cases of hepatic hemangiomas, and twelve cases of normal hepatic tissues were used as the control.

All tissues were scored by two pathologists blinded to disease status. Grading of HCC was based on Edmondson methods [15]. Histopathologic findings of eighty-six HCC samples were divided into four grades according to Edmondson standard, including 18 Grade I, 25 Grade II, 21 Grade III, 22 Grade IV. By the time this study was undertaken, ten patients with HCC had been lost to follow-up or died without known tumor recurrence, and seven patients were excluded who were given post-operative chemotherapy. During the follow-up period, the recurrence-free survival time for the remaining sixty-nine patients was 15.5 months (range 5 to 53 months).

### Immunohistochemical analysis of tissue c-FLIP expression

Sections (4 μm) were deparaffinized, rehydrated, immersed in 3% H_2_O_2 _for 10 min and microwaved at 750 W in citrate buffer (pH 6.0) for 15 min. Tissue sections were then blocked for 20 min with normal rabbit serum and incubated overnight at 4°C with rabbit anti-human c-FLIP polyclonal antibodies, diluted 1:200(Abcan, UK). Incubation with PBS instead of the primary antibody served as a negative control. After washing twice with PBS for 2 min each, immunostaining was performed using the standard S-P technique (Beijing Zhongshan Bio., China) and visualized with diaminobenzidine tetrahydrochloride solution.

Staining was assessed blindly by one observer. A minimum of five randomly selected fields (200×) were examined, with a mean of 1500 cells counted throughout the whole section. The labeling index was defined as the percentage of neoplastic cells with clear cytoplasmic immunoreactivity of the total number of neoplastic cells counted. The threshold for c-FLIP positivity was 10%. The intensity of staining was scored as 0: achromatic, 1: light yellow, 2: yellow, 3: brown.

### Construction of RNAi vectors

According the sequence of the c-FLIP mRNA, the siRNA oligonucleotides were designed and synthesized to the targeted RNAi regions at 526~544, 1164~1182, 1305~1323 nt. *Bgl *II and *Hind *III sites were respectively generated at the 5' and 3' ends of the templates (as shown below). si-526:

5'-CCC**GGAGCAGGGACAAGTTACA**TTCAAGAGA**TGTAACTTGTCCCTGCTCC**TTTTTGGAAA-3' (Forward);

5'-TTTCCAAAAA**GGAGCAGGGACAAGTTACA**TCTCTTGAA**TGTAACTTGTCCCTGCTCC**GGG-3' (Back).

si-1164:

5'-CCC**GCGAGGGCTGTGCACAGTT**TTCAAGAGA**AACTGTGCACAGCCCTCGC**TTTTTGGAAA-3' (Forward);

5'-TTTCCAAAAA**GCGAGGGCTGTGCACAGTT**TCTCTTGAA**AACTGTGCACAGCCCTCGC**GGG-3' (Back).

si-1305:

5'-CCC**ACGCCCACTCCTGGATCTT**TTCAAGAGA**AAGATCCAGGAGTGGGCGT**TTTTTGGAAA-3' (Forward);

5'-TTTCCAAAAA**ACGCCCACTCCTGGATCTT**TCTCTTGAA**AAGATCCAGGAGTGGGCGT**GGG-3' (Back).

These above siRNA-encoding complementary single-stranded oligonucleotides were hybridized to give *Bgl *II- and *Hind *III-compatible overhangs, and then ligated into pSuper (linked overnight at 16°C). After *E. Coli*, DH5α, transfected by the recombinant vectors, the positive clones were selected. With positive plasmids, the sequences were checked by sequencing a PCR-amplified region containing the oligonucleotides. The recombinant plasmids were named as pSuper-Si1, pSuper-Si2, pSuper-Si3 and pSuper-Neg(no target segment inserted), respectively.

### siRNA transfections

HCC cell line, 7721, showed stronger staining intensity (results not shown), and was used as the target cell for the following study. 7721 cells were cultured in RPMI1640(Invitrogen, USA) containing 10% fetal bovine serum(FBS), and maintained in a humidified 37°C incubator with 5% CO_2 _by routine passage every 3 days or as needed.

Plasmids were transfected into cells according to the manufacturer's protocol of LipofectamineTM 2000 (Invitrogen, USA). In brief, 24 hr prior to transfection, cells were seeded without antibiotics in 6-well plate at 3 × 10^5 ^cells/well, corresponding to a density of 80% at the time of transfection. 4 μg plasmids and 8 μL LipofectamineTM 2000 were mixed respectively with RPMI1640 without FBS. These reagents were combined and incubated for 20 min before adding the cells in the mixed liquor. Cells were incubated at 37°C for 8 hr, then fresh RPMI1640 with 10% FBS was added. After another 48 hr cultivation, 400 μg/mL G418 (Promega, USA) was added in. When the cell clones formed after 14 days' growth, cells were screened out to be kept on cultivating. At last, the stable transfection 7721 cell clones were collected and given extended culture.

### RNA preparation and semi-quantitative real-time PCR

Total cellular RNA was extracted from 1 × 10^6 ^cells using TRIzol reagent (Invitrogen, USA). The first strand cDNA was prepared using the Superscript Amplification System kit (Promega, USA) according to the manufacturer's instructions. For PCR, the primer sequences and expected product sizes were as follows: c-FLIP (512 bp), Forward: 5'-ATGTCTGCTGAAGTCAT CC-3', Back: 5'-ATCCTCACCAATCTCCTGCC-3'; β-actin (475 bp), Forward: 5'-TGACGGGGTCACCCACACTGTGCC-3', Back: 5'-CTGCATCCTGTCGGCAATGCCAG-3. Amplification was performed for 25 cycles (15 s denaturing at 95°C, 20 s annealing at 55°C, and 20 s extension at 72°C) in a PERKIN ELMER Thermal Cycler PE2400.

The PCR products were analyzed on 2% agarose gels and visualized by ethidium bromide staining. Quantitation of expression levels was achieved after adjustment for the expression levels of the housekeeping gene β-actin by densitometry (Bio-Rad, USA). The relative level of expression was then represented as the ratio of c-FLIP/β-actin.

### Western Blot Analysis

The transfected 7721 cells were incubated for 30 min at 4°C in lysis buffer [16]. Lysates were cleared at 10,000 × *g *for 10 min at 4°C. Cell lysates were washed three times in cold lysis buffer. 100 μg of total protein was loaded on SDS-polyacrylamide gels, separated by electrophoresis, and transferred to nitrocellulose membranes (Millipore, USA) using standard procedures. The blots were stripped. Blocking of membranes and incubation with the primary (anti-c-FLIP multiclonal Abs) and appropriate secondary Abs were performed. Bands were visualized with an ECL detection kit (Amersham Biosciences, USA).

### Immunocytochemical procedure

Cells were fixed in situ in paraformaldehyde (4% in PBS), and smeared onto slides precoated with 0.01% poly-L-lysine and air dried for 48 hr. Slides were washed in PBS and put into 3% H_2_O_2 _for 15 min to remove endogenous peroxidase activity. Slides were incubated overnight at 4°C with rabbit anti-human c-FLIP polyclonal antibodies. Incubation with PBS instead of the primary antibody served as a negative control. After washing twice with PBS for 2 min each, immunostaining was performed using the standard S-P technique and visualized with diaminobenzidine tetrahydrochloride solution.

### Cell viability assays

For cell viability determination, 2 × 10^4 ^cell/well cell suspension was plated in 96-well microplates. After treated with doxorubicin for 0–8 days, the number of cells per well is obtained by using counting chamber.

### Determination of apoptosis by TUNEL

Cells were treated with the indicated doses of doxorubicin for 48 hr, and then carefully harvested by centrifugation and reattached to gelatin-covered glass slides before labeling. In brief, cells (5 × 10^7^/mL) were fixed in 4% formaldehyde in PBS for 25 min at 4°C. Each glass slide was added 50–100 μL of cell suspension. After air-dry slides at room temperature for 5 min, slides were then washed with PBS for two times. The slides were put into 2% H_2_O_2 _for 5 minutes to remove endogenous peroxidase activity. After removing excess liquid carefully, 50 μL of incubation buffer (45 μL equilibration buffer, 5 μL nucleotide mix containing fluorescein-12-dUTP, and 1 μL terminal deoxynucleotidyl transferase enzyme) were added to each sample. For negative controls: Prepare a control incubation buffer without TdT Enzyme by combining 45 μL of Equilibration Buffer, 5 μL of Nucleotide Mix and 1 μL of autoclaved, deionized water. They were covered with chambered coverslip caps and placed in an incubator under a humidified atmosphere at 37°C for 60 min. Slides were then dipped in stop solution, and incubated 30 min at 37°C. After being washed with PBS at room temperature, the slides were observed under a fluorescence microscope. Apoptosis was indicated by the presence of green or yellow-green fluorescence within the nucleus of cells as confirmation of fluorescein-12-dUTP incorporation at 3'-OH ends of fragmented DNA.

### Statistical analysis

Differences in positive immunostaining rates and expression levels were analyzed by Chi-square test, and comparison of survival curves by Mantel-Cox test, with the software GraphPad Prism 5. The significance was set at P < 0.05.

## Results

### Expression of c-FLIP in human HCC tissues

**Figure 1:**
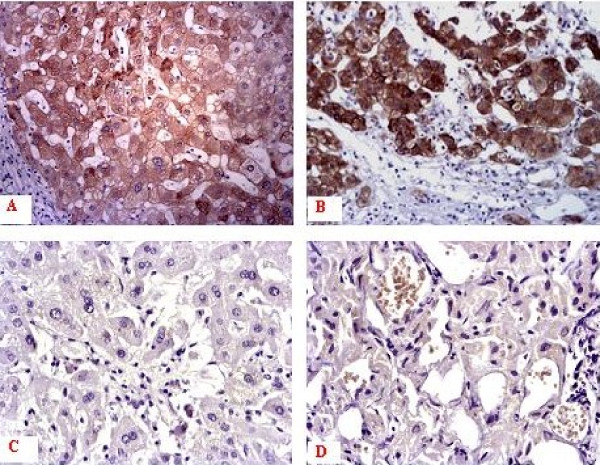
**Expression pattern of c-FLIP in human HCC specimens and corresponding noncancerous liver specimens with anti-c-FLIP antibody**. A: Human HCC specimen with capsular formation; B: HCC specimen with extracapsular invasion; C: Hepatic cirrhosis specimen; D: Hemangioma specimen. (S-P, ×200).

In human HCC tissues, the positive staining showed yellow or brown coloration in the cytoplasm and/or plasma membranes (Figure. 1). Positive human HCC samples displayed stronger staining intensity, compared with the other hepatic samples. Immunoreactivity (defined as expression in 10% or more of neoplastic cells) was detected for c-FLIP in 83.72%(72/86) HCC, 14.81%(4/27) hepatic cirrhosis, 11.11%(2/18) hepatic hemangioma samples, respectively. No immunostaining was found in normal hepatic tissues.

The positive rate in human HCC tissues was related to HCC grade. The rate of c-FLIP positive expression increased with Edmondson standard. The positive expression of c-FLIP displayed in 13/18 (72.22%) samples of Grade I HCC, 20/25 (80.00%) of Grade II, 18/21 (85.71%) of Grade III, and 21/22(95.45%) of Grade IV class (*P *< 0.05). But no correlation was found between the expression of c-FLIP and the tumor stage and size.

**Figure 2:**
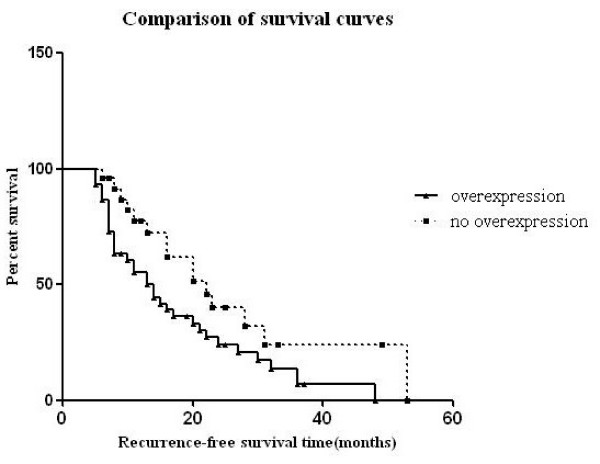
**Recurrence-free survival in relation to c-FLIP expression**. Increased c-FLIP immunoreactivity (c-FLIP overexpression) was associated with shortened survival (Kaplan-Meier curves).

In univariate analysis, c-FLIP expression was not associated with HCC patient survival (*P *= 0.204). But c-FLIP overexpression (more than 50%, *P *= 0.036) implied a lesser probability of survival (Figure. 2). The media recurrence-free survival time for patients with c-FLIP overexpression was 14 months compared with 22 months for those without c-FLIP overexpression.

### Expression of c-FLIP mRNA in different transfected cells

pSuper vector was used for the construction of the recombinant interfering vectors. DNA sequencing of the plasmids verified the successful construction of the c-FLIP RNAi vectors. The three positive plasmids were termed as pSuper-Si1, pSuper-Si2, and pSuper-Si3, containing the distinct siRNA segment respectively. pSuper-Neg, without the interfering segment, was used as the control.

**Figure 3:**
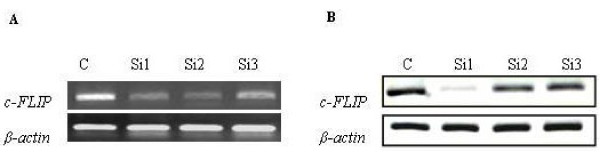
**Expression of c-FLIP mRNA and protein in the transfected cells**. A: c-FLIP mRNA. B: c-FLIP protein. (C: control cells transfected by pSuper-Neg; Si1: 7721 cells transfected by pSuper-Si1; Si2: 7721 cells transfected by pSuper-Si2; Si3: 7721 cells transfected by pSuper-Si3;)

We examined expression levels of c-FLIP mRNA in the transfected cells with different recombinant vectors (named 7721/pSuper-Si1, 7721/pSuper-Si2, 7721/pSuper-Si3 and 7721/pSuper-Neg, respectively), using a semi-quantitative RT-PCR assay. The comparable amplification efficiencies were validated by the uniformity of control β-actin RT-PCR product yields. RT-PCR results showed that the expression levels of c-FLIP mRNA were inhibited in the transfected cells (Figure. 3A), but the expression levels varied between these cells. c-FLIP mRNA expression in 7721/pSuper-Si1 cells was significantly lower than that in the other two transfected cells.

Then we examined the effect of siRNA on the expression of c-FLIP protein with Western Blot and immunocytochemical staining. First, c-FLIP protein expression was analyzed by Western blot analysis (Figure. 3B). pSuper-Si1 obviously decreased the expression of c-FLIP protein. The results supported the fact that si-526-siRNA inhibited c-FLIP expression specifically. To further evaluate the effect of siRNA, we studied the c-FLIP protein expression by immunocytochemical staining. Immunocytochemical analysis showed that the primary 7721 cells were strongly immunostained with the anti-c-FLIP antibodies, compared to 7721/pSuper-Si1.

### Doxorubicin-induced apoptosis inhibited by siRNA

**Figure 4:**
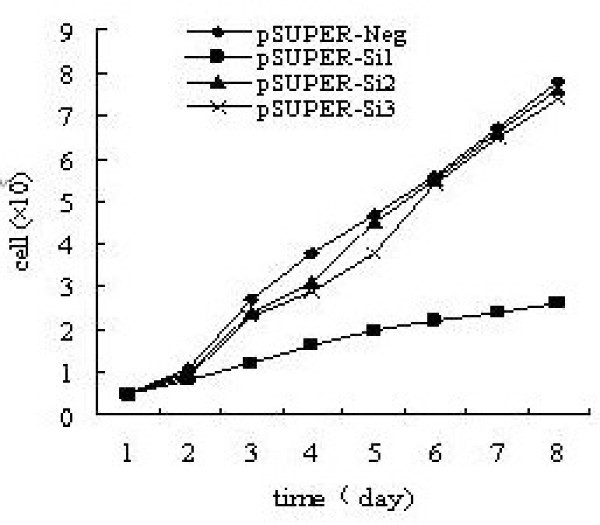
**Cell viability was accessed by cell counting**. The study showed that 7721 cell viability was reduced by the transfetion with recombinant iRNA vectors. pSuper-Si1 had more significant effect on the reduction of the cell viability.

Cells were treated with doxorubicin for 0–8 days, and then the growth curves were obtained. Cell proliferation was inhibited obviously when c-FLIP expression was knocked down by siRNA. Our data showed that si-526-siRNA significantly decreased the growth rate of 7721 cells, with a >50% decrease after 3 days repeatedly in three separate experiments (Figure. 4).

**Figure 5:**
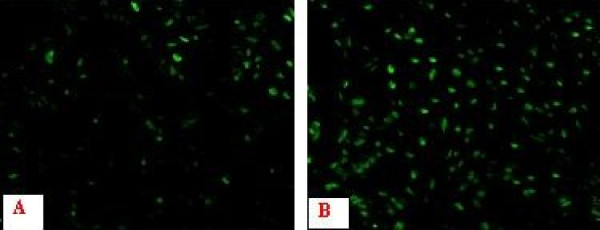
**Cells were assayed for apoptosis by the TUNEL method and photographed by fluorescence microscopy at ×100**. Green cells are positive for DNA fragmentation, consistent with apoptosis. A: 7721/pSuper-Neg; B: 7721/pSuper-Si1.

Then, the cells were assayed by the TUNEL method to assess the drug-induced apoptosis. Positive TUNEL staining would be indicative of the DNA fragmentation that was characteristic of apoptosis. Without c-FLIP RNAi, the fewer 7721 cells were TUNEL positive. In contrast, in cells transfected with the specific siRNA vector, pSuper-Si1, the apoptosis induced by treatment with doxorubicin was significantly elevated (Figure. 5).

## Discussion

Tumor cells have developed different ways to escape apoptosis induced by DR-triggering such as surface DR down-regulation, loss or mutation. Other mechanisms elaborated by tumor cells to develop cell death resistance include aberrant expression of anti-apoptotic molecules such as c-FLIP, Bcl-2, Bcl-xL, survivin and Livin. The current belief holds that perturbations in apoptotic death regulation constitute a vital step in cancer evolution [17].

Each step in DR-mediated apoptosis is well regulated. c-FLIP is a recently identified intracellular inhibitor of caspase-8 activation that potently inhibits death signaling mediated by all known death receptors, including Fas, TNF-receptor (TNF-R), and TNF-related apoptosis-inducing ligand receptors (TRAIL-Rs). Furthermore, c-FLIP over-expression can activate nuclear factor (NF)-κB activation induced by TNF-α or TRAIL. c-FLIP has a more central role in the antiapoptotic NF-kB response than the TRAF/IAP complex. On the other hand, c-FLIP expression is regulated by NF-κB and phosphatidylinostiol-3 kinase (PI-3)/Akt pathways. So, c-FLIP plays an important role in cell survival not simply by inhibiting DR-mediated apoptosis but also by regulating NF-κB activation in human HCCs [10,18]. Moreover, c-FLIP has recently been shown to be associated with the generation of positive signals for cell proliferation by activation of the Erk pathway through Raf-1 binding [19,20].

There is increasing evidence that in regard to its anti-apoptotic functions, c-FLIP can be considered as a tumor-progression factor. At present, the role of c-FLIP, as an anti-apoptotic protein involved in the regulation of the DR extrinsic apoptotic pathway, remains unclear. Therefore, the identification of the precise role of c-FLIP in cancer cells represents an essential aim to further target and restore deregulated death pathways.

c-FLIP is generally expressed in embryonic tissues, but is not expressed in most normal adult tissues, whereas is over-expressed in the majority of human cancers. It indicates that c-FLIP may associate with the tumorigenesis and progress of most human cancers. Published information regarding the significance of c-FLIP over-expression in human tumors has only recently begun to accumulate [21-24].

Human HCCs show resistance to apoptosis mediated by several death receptors. c-FLIP is constitutively expressed in human HCC cell lines, and is expressed with a higher positive rate in human HCC tissues than in noncancerous liver tissues. In the present study, positive immunostaining was detected for c-FLIP in 83.72% of human HCC samples, but was absent from normal hepatic tissues. The other authors' and our studies suggest that c-FLIP may play an important role in human HCCs. For the patients with c-FLIP overexpression, they may have a shorter recurrence-free survival time.

Now, RNAi, that can induce highly specific target gene silencing in mammalian cells using siRNA, has been a powerful tool in studying the cell function of any gene. c-FLIP expression can be inhibited by RNA interference using siRNAs, evidence from reduced levels of c-FLIP mRNA and c-FLIP protein[25].

In this study, the c-FLIP-targeted siRNA vectors were designed to specifically silence c-FLIP. Then, the plasmids transcript containing c-FLIP-targeted siRNA and negative siRNA were constructed and transfected into 7721 cells. We found that there were significant differences between 7721/pSuper-Si1 and 7721/pSuper-Neg in c-FLIP expression at both mRNA and protein levels (Figure. 3A, Figure. 3B). The phenomenon that screened positive clone with lower c-FLIP expression indicated that the c-FLIP-targeted siRNA inhibited c-FLIP expression specifically.

Some studies reported that siRNA-mediated silencing of c-FLIP induced spontaneous apoptosis in a panel of p53 wild-type, mutant, and null colorectal cancer cell lines [11]. And the anti-apoptotic role of c-FLIP in regulating TRAIL-mediated apoptosis in colon cancer cells was clearly shown using siRNA methodology [26]. Furthermore, c-FLIP down-regulation sensitized colorectal cancer cells to chemotherapy [27]. And, specific silencing of c-FLIP_L _was sufficient to sensitize MDA435 cells to doxorubicin. Our study showed that c-FLIP gene silencing enhanced doxorubicin-induced HCC cell apoptosis (Figure. 5). These results indicate that c-FLIP may be an important regulator of chemotherapy-induced cell death in human HCC cells.

## Conclusion

The results of the present investigation demonstrated that c-FLIP is frequently expressed in human HCCs, correlated with Edmondson standard. The HCC patients with c-FLIP overexpression may have a shorter recurrence-free survival time. The specific silencing of c-FLIP can apparently inhibit its expression on both protein and mRNA levels. The inhibition of c-FLIP expression can down-regulate HCC cell viability and up-regulate drug-induced cell apoptosis. Our data suggest that targeting c-FLIP in conjunction with anticancer therapies may have therapeutic potential by enhancing HCC cell death.

## Competing interests

The authors declare that they have no competing interests.

## Authors' contributions

XD and GB participated in the preparation of the tissue sections and the construction of the siRNA vectors, and helped in coordinating the work. GB also participated in data analysis and interpretation and in manuscript preparation. XH and QQ have been involved in western blot analysis, PCR assays and IHC and ICC assays of c-FLIP expression. HZ and FY participated in cell culture and cellular work. JL participated in study design and critical revision of the manuscript. QM participated in the study design and coordination and helped to revise the manuscript. All authors read and approved the final manuscript.
